# Two-Dimensional Zymography Differentiates Gelatinase Isoforms in Stimulated Microglial Cells and in Brain Tissues of Acute Brain Injuries

**DOI:** 10.1371/journal.pone.0123852

**Published:** 2015-04-10

**Authors:** Shanyan Chen, Fanjun Meng, Zhenzhou Chen, Brittany N. Tomlinson, Jennifer M. Wesley, Grace Y. Sun, Adam T. Whaley-Connell, James R. Sowers, Jiankun Cui, Zezong Gu

**Affiliations:** 1 Department of Pathology and Anatomical Sciences, University of Missouri School of Medicine, Columbia, Missouri, United States of America; 2 Center for Translational Neuroscience, University of Missouri School of Medicine, Columbia, Missouri, United States of America; 3 Interdisciplinary Neuroscience Program, University of Missouri, Columbia, Missouri, United States of America; 4 MS in Pathology program, University of Missouri Graduate School, Columbia, Missouri, United States of America; 5 Department of Biochemistry, University of Missouri School of Medicine, Columbia, Missouri, United States of America; 6 Department of Internal Medicine Diabetes and Cardiovascular Center, University of Missouri School of Medicine, Columbia, Missouri, United States of America; 7 Harry S. Truman Memorial Veterans’ Hospital, Columbia, Missouri, United States of America; INRS, CANADA

## Abstract

Excessive activation of gelatinases (MMP-2/-9) is a key cause of detrimental outcomes in neurodegenerative diseases. A single-dimension zymography has been widely used to determine gelatinase expression and activity, but this method is inadequate in resolving complex enzyme isoforms, because gelatinase expression and activity could be modified at transcriptional and posttranslational levels. In this study, we investigated gelatinase isoforms under in vitro and in vivo conditions using two-dimensional (2D) gelatin zymography electrophoresis, a protocol allowing separation of proteins based on isoelectric points (pI) and molecular weights. We observed organomercuric chemical 4-aminophenylmercuric acetate-induced activation of MMP-2 isoforms with variant pI values in the conditioned medium of human fibrosarcoma HT1080 cells. Studies with murine BV-2 microglial cells indicated a series of proform MMP-9 spots separated by variant pI values due to stimulation with lipopolysaccharide (LPS). The MMP-9 pI values were shifted after treatment with alkaline phosphatase, suggesting presence of phosphorylated isoforms due to the proinflammatory stimulation. Similar MMP-9 isoforms with variant pI values in the same molecular weight were also found in mouse brains after ischemic and traumatic brain injuries. In contrast, there was no detectable pI differentiation of MMP-9 in the brains of chronic Zucker obese rats. These results demonstrated effective use of 2D zymography to separate modified MMP isoforms with variant pI values and to detect posttranslational modifications under different pathological conditions.

## Introduction

Matrix metalloproteinases (MMPs) are a family of 26 zinc-dependent endopeptidases that possess structurally similar hemopexin, propeptide, and catalytic domains. Even though MMPs are involved in cell remodeling and dynamical homeostasis during development, aberrant regulation and activity of MMPs, particularly the gelatinase (MMP-9/2), have also been shown in pathological conditions, including angiogenesis in cancer, disruption of the blood—brain barrier, as well as neuroinflammation in stroke and traumatic brain injury (TBI) [1–7]. There is sufficient evidence indicating that gelatinases can generate autoimmunity and skew immune functions, such as cleaving myelin basic protein, type II gelatins, as well as cytokines and chemokines into remnant fragments [8]. Dysregulation of immune homeostasis can lead to autoimmune diseases such as multiple sclerosis, rheumatoid arthritis and diabetes [9,10]. Under these pathological conditions, MMPs can be regulated at different levels, including transcriptional [11] and posttranslational modifications, and inhibition of enzymatic activity by endogenous tissue inhibitors of metalloproteinases (TIMP). In particular, MMPs have been shown to undergo posttranslational modifications by peroxynitrite-induced protein *S*-glutathiolation *in vitro* [12] and by nitric oxide (NO)-mediated protein *S*-nitrosylation after cerebral ischemia [13].

Microglia are innate immune cells that play important roles in responding to oxidative and inflammatory stress and to different forms of brain insults [14], such as increased microglial activation in mice with transient bilateral common carotid artery occlusion [15]. Microglia can be activated and change from a ramified to an amoeboid morphology in response to toxins and proinflammatory cytokines [16] or after traumatic injury [17]. The murine BV-2 microglial cells have been used as cell models to elucidate proinflammatory signaling pathways and responses of MMPs to endotoxin lipopolysaccharide (LPS) [18,19]. Increase in MMP-9 together with microglia activation is associated with a number of neurodegenerative diseases. Microglial activation is associated with induction of NADPH oxidase, an enzyme complex that generates reactive oxygen species [20–22].

The neurovascular unit is comprised of the cerebral vascular endothelium, tight junctions, astrocytes, pericytes, neurons, and the extracellular matrix, and is essential for the health and function of the central nervous system (CNS) [23]. Excessive upregulation of MMPs, especially the gelatinases, has been implicated in the disruption of cerebrovasculature in neurodegenerative diseases, such as cerebral ischemia [24] and TBI [25]. There is evidence that diabetic obesity is associated with both vascular impairment and neurodegenerative conditions [26], and MMP-9 upregulation in diabetic mice exacerbates white matter damage after stroke [27].

Substantial evidence indicates posttranslational and posttranscriptional modifications of gelatinases in pathological conditions and in response to pro-inflammatory cytokines. In mouse calvarial cultures, the levels of both pro- and active forms of gelatinases were increased in the conditioned medium upon induction by IL-1, a pro-inflammatory cytokine [28]. In another study, posttranslational modification was observed upon exposing HT cells to PKC activator and by reacting purified MMP-2 with PKC [29]. These results suggest that inflammation-induced transcriptional and posttranslational modifications can be a key mechanism for upregulation and activation of gelatinases under pathological conditions.

Zymography is a highly sensitive method used to detect protease activities by visualization of the conversion of their substrates [30]. This method can differentiate proteins based on their molecular weights by electrophoresis under non-reducing conditions, and followed by reactivation of the enzymes and visualization of the proteolytic activity of gelatinases by staining with Coomassie blue. In this study, we applied a two-dimensional (2D) gelatin zymography technique that combines isoelectric focusing (IEF) with zymographic electrophoresis to achieve significant improvement in separation of the enzymatic isoforms in various pI values. Using this assay protocol, we detected modifications of gelatinase isoforms with different pI values, and observed differences in gelatinase isoforms in cell and brain tissues from rodent models subjected to acute brain injuries and chronic metabolic disorder.

## Results

### Gelatinase isoforms identified from HT1080 cell conditioned medium by 2D zymography

**Fig 1:**
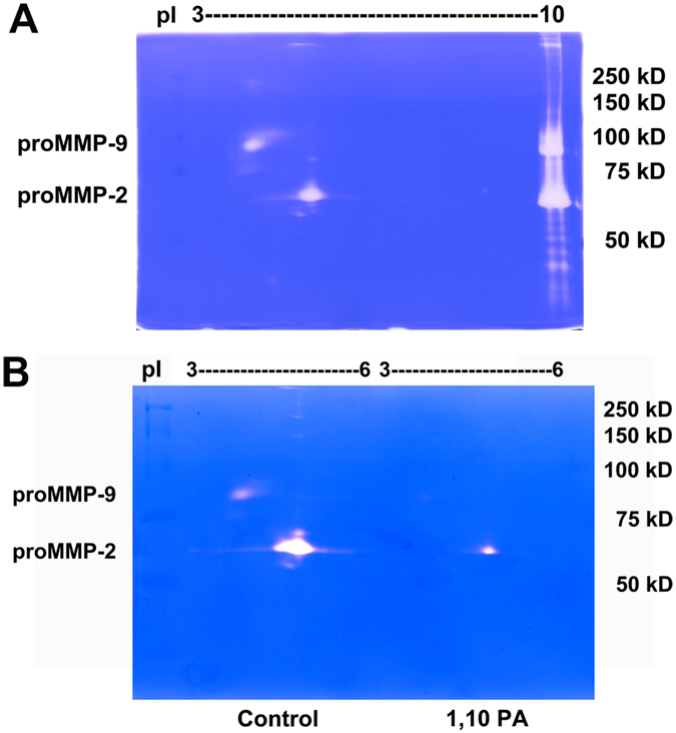
Identification of gelatinase isoforms from HT1080 cell conditional medium by 1D and 2D zymography. After incubating with gelatin Sepharose 4B (gelatin 4B) overnight in conditioned medium of HT1080 cells 1D and 2D zymography was performed. Transparent spots (2D) and bands (1D, right side of the gel) revealed proteolytic activity of gelatinases. (**A**) 2D zymogram showed a 92-kDa proMMP-9 spot with pI value between 3 and 4, and a 65-kDa proMMP-2 spot with pI value between 4 and 5, corresponding to the respective molecular weights of the bands resolved by 1D zymography on the right of the same gel. (**B**) Conditioned medium of HT1080 cells treated with or without a broad-spectrum MMP inhibitor 1,10 PA were loaded to two IEF dry strips. After IEF separation, these two strips were cut in the pI values ranging from 3 to 6 based on the 2D zymography mapping in Fig 1A, then loaded on the same SDS-PAGE gel for comparison. MMP inhibitor 1,10 PA attenuated gelatinase activity compared to the untreated control.

To determine if various enzymatic patterns, including enzyme posttranslational isoforms, affect enzymatic activity, non-reducing 2D zymography was performed. In this study, gelatinases present in the conditioned medium of HT1080 cells (human fibrosarcoma cells with abundant gelatinases) were enriched with gelatin Sepharose 4B (gelatin 4B) and subsequently analyzed using 1D and 2D zymography. Gelatinases separated by different pI values and molecular weights were visualized as transparent spots on Coomassie blue-stained gels. A 92-kDa proform MMP-9 (proMMP-9) spot with pI value between 3 and 4, and a 65-kDa proMMP-2 spot with pI value between 4 and 5 were identified (Fig 1A). The broad-spectrum MMP inhibitor 1,10 phenanthroline (1,10 PA), which was exposed to the enriched HT1080 cell condition medium, attenuated the intensity of MMP-9 and MMP-2 spots (Fig 1B), confirming the presence of MMP-2 and MMP-9 with different pI values. To test the sensitivity of gelatinase activity, 1–2 ng of purified MMP-9 was analyzed using 1D and 2D zymography with overnight incubation at 37°C, and displayed intensive spots with gelatinase activity (S1 Fig). From these *in vitro* studies, we demonstrated 2D zymography as an effective method for determination of sub-nanogram quantities of gelatinases with various pI values.

**Fig 2:**
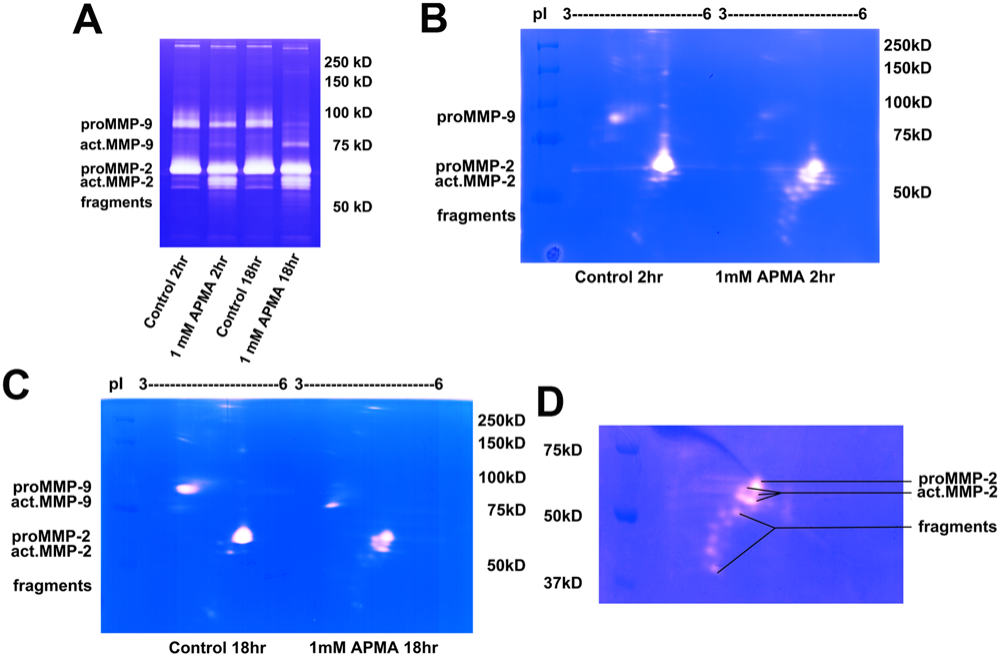
2D zymography reveals gelatinase isoforms from HT1080 cell conditioned medium *in vitro* exposed to APMA. After incubating with conditioned medium of HT1080 cells, gelatin 4B were exposed to 1 mM APMA, 2 h for MMP-2 activation or 18 h for MMP-9 activation. (**A**) 1D gelatin zymography revealed gelatinase activity after exposure to APMA. (**B**, **C**) Gelatinase isoforms were detected using 2D zymography. Samples with 2-h and 18-h incubation with or without APMA were loaded to IEF dry strips. After IEF separation, strips were cut in the pI values ranging from 3 to 6 and loaded on the same SDS-PAGE gel for comparison, respectively. (**D**) Enlarged photograph of the 2D zymogram displayed 4 isoforms and multiple fragments of active MMP-2. These zymograms are representative results from 4 independent experiments.

2D zymography was used to analyze the HT1080 conditioned medium after the enriched conditioned medium was exposed to organomercury 4-Aminophenylmercuric acetate (APMA), an agent known to activate gelatinases. Because kinetics for *in vitro* activation of MMP-2 and MMP-9 may be different, HT1080 conditioned medium was exposed to APMA for 2 h or 18 h accordingly. At 2 h after exposure to APMA, gelatinases were separated into four main bands―proMMP-9, proMMP-2, active MMP-2 (act.MMP-2) and fragments (Fig 2A). After 18 h exposure, proMMP-9 was activated into the active form with lower molecular weight. Interestingly, after APMA activation, 2D zymography for proMMP-9 and proMMP-2 still showed one transparent spot each, whereas a number of spots can be identified as act.MMP-2 at 60 kDa and seven spots identified as MMP-2 fragments with pI variants (Fig 2B). Activated MMP-9 (act.MMP-9) did not show pI changes (Fig 2C). In the close-up region of gelatin zymogram (Fig 2D), four main spots of act.MMP-2 had similar molecular weights, but were separated by their different pI values. These isoforms were not able to be distinguished by the conventional 1D zymography protocol.

### Gelatinase isoforms from the conditioned medium of LPS-stimulated BV-2 microglial cells

**Fig 3:**
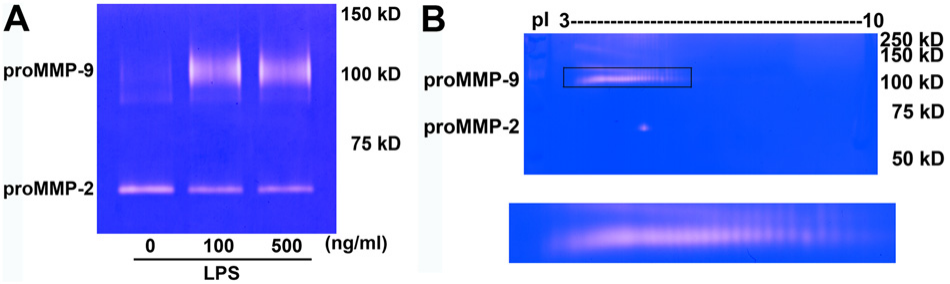
2D zymography reveals gelatinase isoforms from LPS-stimulated microglial BV-2 cells conditioned medium. (**A**) BV-2 cells were treated with 100 or 500 ng/ml endotoxin lipopolysaccharide (LPS) for 16 hr. Proteolytic activity of gelatinases was visualized as a single band of proform of gelatinases (proMMP-2 and proMMP-9) by 1D zymography. (**B**) In 2D zymograms, proMMP-9 isoforms were visualized as a serious 105-kDa transparent spots with pI values ranging from 3.5 to 7 and proMMP-2 as a 65-kDa single spot with pI value between 4 and 5. These zymograms are representative results from 3 independent experiments.

In this study, we further examined gelatinase activity, this time in BV-2 microglial cells upon stimulation with LPS. 1D zymography indicated the presence of proMMP-9 bands in the conditioned medium after exposing BV-2 cells to LPS (100 or 500 ng/mL) for 16 h (Fig 3A). In 2D zymography, proMMP-9 was visualized as a series of spots with pI values ranging from 3.5 to 7 (Fig 3B), compared to a single spot detected from the HT1080 conditioned medium (Fig 1A). MMP-2 did not display a pI shift.

**Fig 4:**
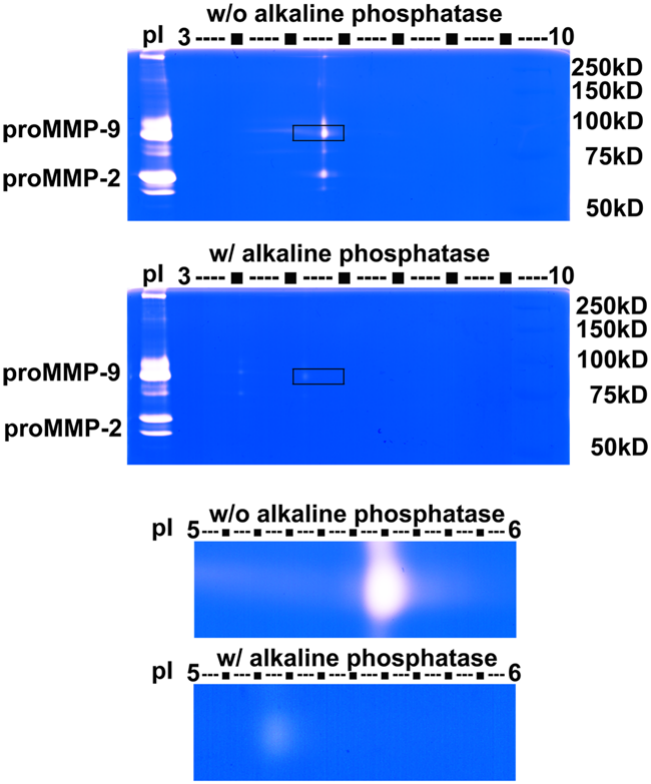
2D zymography detects phosphorylation of MMP-9 isoforms from LPS-stimulated microglial BV-2 cells conditioned medium. BV-2 cells were treated with 100 ng/mL LPS for 16 h. Conditioned medium was enriched by gelatin 4B, incubated with 10 unit alkaline phosphatase (37°C, 1 h), and then analyzed by 2D zymography. Central spot of MMP-9 shifted from pI 5.60 to 5.25 after alkaline phosphatase treatment. These zymograms are representative results from 3 independent experiments.

Because LPS is known to stimulate a number of protein kinases in microglial cells, it is reasonable to examine whether changes in the pI shifts of MMP-9 after LPS stimulation is due to phosphorylation of the protein. Conditioned medium from LPS-stimulated BV-2 cells was concentrated with gelatin 4B and then subjected to dephosphorylation by incubation with alkaline phosphatase. After treatment with alkaline phosphatase, pI value of the central spot of MMP-9 isoform was shifted from approximate 5.60 to 5.25 with large reduction in gelatinase activity (Fig 4). These results suggest phosphorylation occurred in MMP-9 isoforms associated with increase in LPS-induced enzymatic activity.

### Gelatinase isoforms identified from mouse brain tissue after focal cerebral ischemia

**Fig 5:**
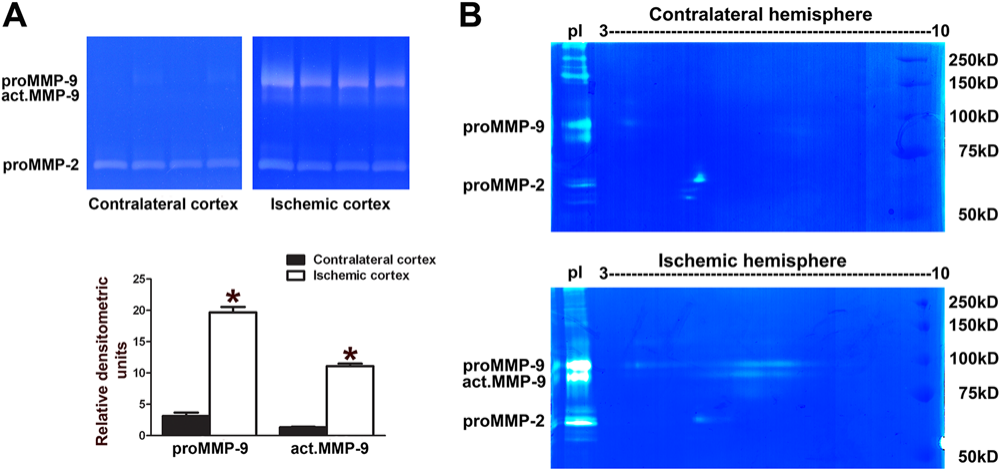
2D zymography reveals gelatinase isoforms in mouse brains after focal cerebral ischemia. Mice were sacrificed 24 h after filament-induced MCAo in mice. (**A**) 1D zymography revealed increases in proMMP-9 and act.MMP-9 levels in the ischemic cortex compared with the contralateral cortex. Under these experimental conditions, proMMP-2 was not altered. Densitometry analysis of intensity of gelatinolytic bands represented proMMP-9 and act.MMP-9, n = 4, *, *p*<0.001, comparing ischemic to contralateral cortex by one-tailed, unpaired Student’s *t*-test; data are expressed as mean values ± SEM. (**B**) Brain lysate was incubated with gelatin 4B and applied on 2D gels. In contralateral hemispheres, proMMP-9 was identified as a 105-kDa single spot with pI value between 3 and 4, and proMMP-2 as a 65-kDa single spot with pI value between 4 and 5. In ischemic hemispheres, two streaks of pI values ranging from 5.5 to 8 with the molecular weights at 105 and 95 kDa were identified as proMMP-9 and act.MMP-9, respectively. Ischemic brain lysate was applied on the left side of the gel for comparison. These zymograms are representative results from 3 independent experiments.

Excessive MMP-9 activity is known to be involved in brain injury such as stroke, and exacerbates neuronal apoptosis and impairment of neurovasculature. In this experiment, we used the focal cerebral ischemia model in mice induced by filament insertion to occlude the middle cerebral artery of mice (MCAo). Quantification of gelatinase activity in the ischemic cortex 24 h after MCAo showed a significant increase in levels of both proMMP-9 and act.MMP-9 in the ischemic cortex as compared to the contralateral region (Fig 5A). In 2D zymography, a 105-kDa proMMP-9 spot with pI value between 3 and 4 was observed in the contralateral hemisphere (Fig 5B). In contrary, an additional streak of proMMP-9 at 105-kDa and an additional streak of act.MMP-9 at 95-kDa were identified in the ischemic hemisphere. Both of the streaks had pI values ranging from 5.5 to 8.0.

### Neuroinflammatory responses in mouse brain after focal ischemia

**Fig 6:**
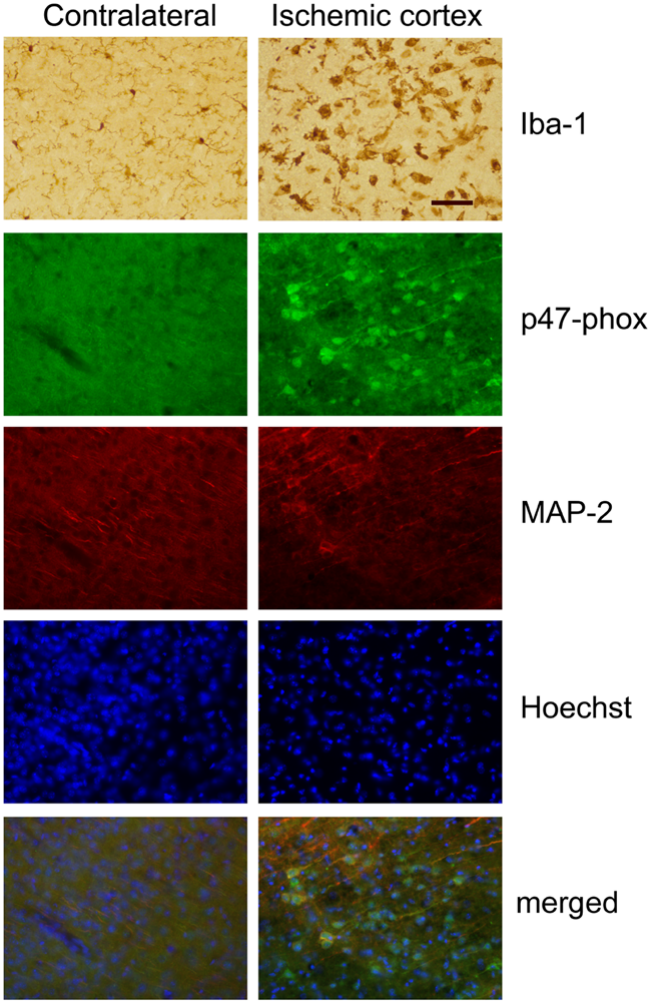
Microglial activation and neuronal cell death in ischemic cortex. Representative photomicrographs of mouse cortical region of brain sections stained with microglia marker Iba-1, NADPH oxidase maker p47phox, and neuron marker MAP-2. Nuclear DNA was counterstained with Hoechst dye. Scale bar, 50 μm.

We next investigated microglia and NADPH oxidase activation, and neuronal cell death after MCAo using immunofluorescent staining. The amoeboid form of activated microglia and upregulation of the NADPH oxidase subunit p47phox were observed in the ischemic cortex, but not in the contralateral cortex (Fig 6). Neuronal cell death and dendritic degeneration were observed in ischemic cortex, indicating that there was proinflammatory response-induced neuronal cell death in the ischemic cortex.

### Gelatinase isoforms from mouse brain after TBI

**Fig 7:**
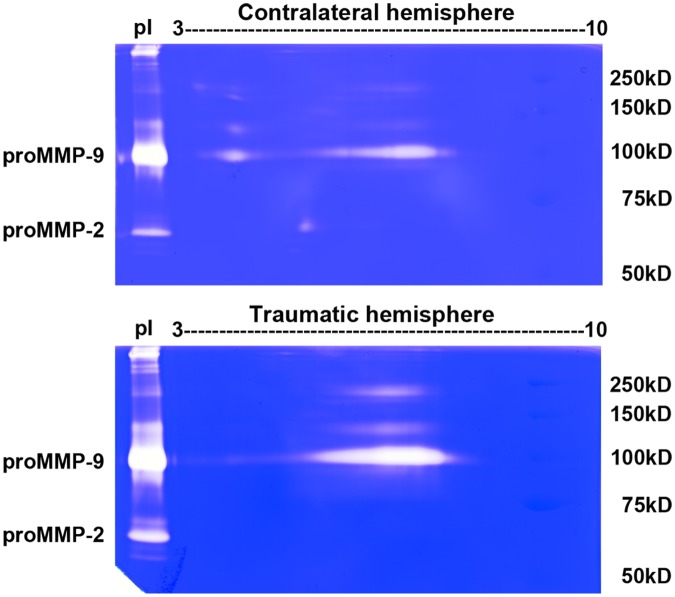
2D zymography reveals enzymatic isoforms of gelatinases in TBI mouse brains. Mice were sacrificed 6 h after CCI-induced TBI. In contralateral hemispheres, proMMP-9 was identified as a 105-kDa single spot with pI value between 3 and 4 and a 105-kDa streak of pI values ranging from 5.5 to 8. In lesioned hemispheres, proMMP-9 was identified as a streak with higher intensity of pI values ranging from 5.5 to 8. Traumatic brain lysate was applied on the left side of the gel for comparison. These zymograms are representative results from 4 independent experiments.

TBI has been shown to cause increases in MMP-9 in the brain. Using the controlled cortical impact model, 2D zymography showed proMMP-9 as a single spot at 105-kDa with pI value between 3 and 4, and a streak with pI values ranging from 5.5 to 8 in the contralateral hemisphere at 6 h after injury (Fig 7). In the lesioned hemisphere, the intensity of MMP-9 streak was much higher, and the MMP-9 spot with pI between 3 and 4 was not observed.

### Increased levels of MMP-9, but not its isoforms, in brain tissues of rats with chronic diabetic obesity

**Fig 8:**
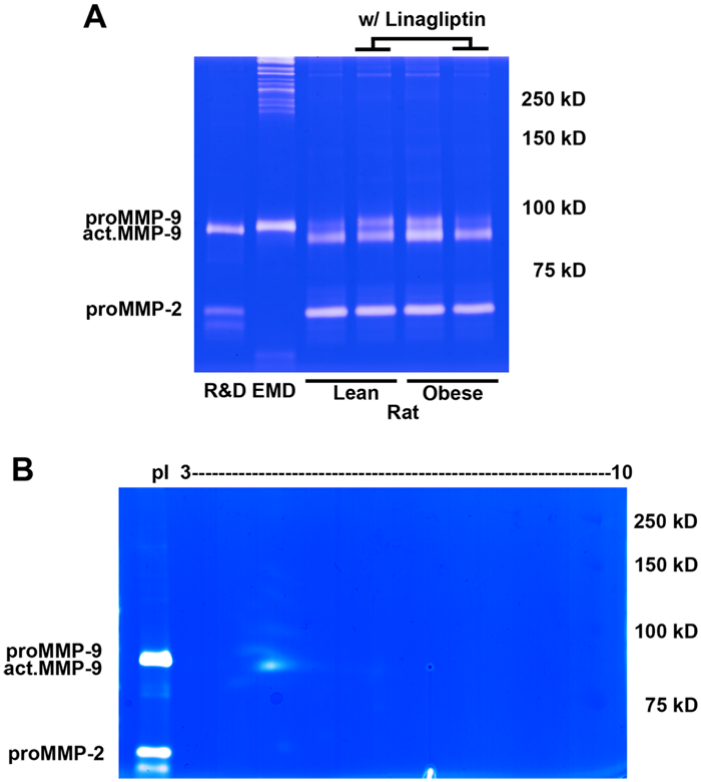
2D zymography reveals enzymatic isoforms of gelatinases of brain tissues from ZO rats. (**A**) Gelatinase activity from the brain tissues of the Zucker lean and obese rats was visualized using 1D gelatin zymography. ProMMP-9, act.MMP-9 and proMMP-2 were identified as bright bands. For the act.MMP-9, ZO vehicle-treated rats showed stronger bands than Zucker lean rats; Linagliptin ameliorates MMP-9 upregulation. (**B**) Gelatinase isoforms from ZO rats were visualized with 2D gelatin zymography. ProMMP-9 was identified as a 105-kDa single spot, act. MMP-9 as a 95-kDa single spot both with pI values between 3 and 4, as well as proMMP-2 as a 65-kDa single spot with pI value between 4 and 5. Purified gelatinases were applied on 1D zymography on the left side of the same gel for comparison. These zymograms are representative results from 4 independent experiments.

To examine whether the posttranslationally modified MMP-9 isoforms are also involved in chronic metabolic disease, we examined cortex of the Zucker obese (ZO) rats, a rodent model of chronic diabetic obesity. Results with 1D zymography showed higher levels of act.MMP-9 in the ZO rats compared to the Zucker lean rats. In addition, levels of MMP-9 were reduced after treatment of linagliptin, an inhibitor for dipeptidyl peptidase-4 enzyme to attenuate aberrant biosynthesis and secretion of insulin to treat Type 2 diabetes (Fig 8A). With 2D zymography, proMMP-9 and act.MMP-9 were clearly separated (Fig 8B), and no pI shift of MMP-9 was detected in the ZO rats as compared to the changes induced after acute ischemic and traumatic injuries. No pI shifts of MMP-9 were found in 2D zymography of Zucker lean rats and ZO rats after treatment of linagliptin (data not shown).

## Discussion

MMPs are important enzymes required to mediate tissue homeostasis. Though most MMPs are secreted or membrane-bound as inactive zymogens [31], posttranslational modifications have been shown as important factors in regulating the functional activities of MMP-2 [29]. Posttranslational modifications such as glycosylation, sialylation, nitrosylation, and phosphorylation of gelatinases have been demonstrated as important in regulating their functional activities [13,29,32,33]. Since posttranslational modifications of proteins result in a pI shift, identification of modification isoforms of MMPs may provide mechanistic insight into the roles of gelatinases in pathological processes of neurodegenerative diseases.

Gelatin zymography is an established, simple and sensitive assay to analyze MMP activity in biological samples [34]. However, identification of isoforms of gelatinases based on the molecular weight by 1D zymography does not differentiate the gelatinase pI variants caused by posttranslational modifications. 2D polyacrylamide gel electrophoresis (PAGE) is one of the most common methods to visualize protein isoforms with pI shift [35]. However, because of the very low stoichiometry of enzyme posttranslational modifications in *in vivo*, the modification isoforms of MMPs may remain undetected on the conventional 2D gels. 2D zymography combines 2D PAGE and zymography providing two-dimensional separations and thus help to resolve the complex isoforms of MMPs with high sensitivity. 2D zymography was first introduced to detect posttranslational modifications of gelatinases in explanted hearts from coronary heart disease patients [36], and was recently reported to identify heterogeneous isoforms of gelatinases and their charge variants in sera of patients with multiple sclerosis [33].

In this study, we used 2D zymography to analyze activity of gelatinases enriched from conditioned medium of HT1080 cells. Our results showed a number of modification isoforms with different pI values, suggesting that 2D zymography is an effective assay to detect modification isoforms of gelatinases. Our results are consistent with other reports about changes in protease isoforms from green kiwi fruit [37], bacillus [38], snake venom [39,40], marine sponges [41] and Atlantic cod muscle [42]. Oxidative stress, such as peroxynitrite, is known to be involved in the activation of MMPs through a mechanism that does not involve the proteolytic removal of the inhibitory pro-peptide domain [12,43]. Therefore, detection of proform and active form of MMPs based solely on molecular weight identification by 1D zymography may not be effective for providing results of protein modifications. Increased production of MMP-9 has been shown in rodent lung fibroblasts, epithelial cells and macrophages upon stimulation with proinflammatory mediators, including LPS [44,45]. In this study, we applied 2D zymography to LPS-stimulated microglial cells and rodent models of acute brain ischemic and traumatic injuries, as well as rat brains of chronic diabetic obesity.

Modification of isoforms of gelatinases s was observed in BV-2 cells stimulated with LPS. We observed a series of spots with the same molecular weight, consistent with proMMP-9, with pI values ranging from 3.5 to 7. These findings suggest that posttranslational modifications of MMP-9 occurred in microglia upon stimulation with LPS. There are reports that MMP-2 phosphorylation modulates its proteolytic activity in the human fibrosarcoma cell line and detection of heterogeneous isoforms of gelatinases in sera of multiple sclerosis patients [29,33]. In this study, the pI value of the major MMP-9 isoform was shifted from 5.60 to 5.25 with reduced gelatinase activity after treatment with alkaline phosphatase, an enzyme known to dephosphorylate proteins. These results also suggest the significance of phosphorylation of MMP-9 isoforms in maintaining enzymatic activity.

Excessive increase in gelatinases associated with inflammatory responses in neurodegenerative diseases is well documented [3,46]. In our previous study, we observed a dramatic increase in levels of proMMP-9 and act.MMP-9 in the ischemic cortex within 24 h after focal cerebral ischemia, with no significant changes in MMP-2 levels [24,47]. In this study, we found that the proMMP-9 spot with pI between 3 and 4 was in the contralateral hemisphere. In contrast, the proMMP-9 and act.MMP-9 streaks of pI values ranging between 5.5 and 8.0 were identified in the ischemic hemisphere. We also found pI shift of MMP-9 in the lysates of the lesioned hemispheres from the mice with TBI. The streak shown in the contralateral hemisphere was likely due to the of 15° angle at which the trans-hemispheric controlled cortical impact (CCI) was induced, causing a countercoup injury. As in our previous study, we observed a dramatic increase in levels of proMMP-9 and act. MMP-9 in the lesioned cortex within 24 h and remaining elevated at 10 days after TBI with no significant changes in MMP-2 levels [48]. Studies with 2D zymography further indicated changes in isoforms of MMP-9 with pI variants in neuroinflammation in microglial cells and in the models of acute brain injuries.

Obesity is considered to be a chronic, low-grade inflammatory stress [49]. Metabolic disorders are associated with type 2 diabetes which may involve in dysregulation of neurophysiology resulting in neurodegeneration [50,51]. The Zucker rats have been reported to have insulin resistance [52], distal degenerative sensory neuropathy [53], and increased oxidative and nitrosative stress [54]. In 2D zymograms of ZO rats, the proMMP-9 and act.MMP-9 were identified as single spots with pI between 3 and 4, similar to those observed in the HT1080 *in vitro* activation of 2D zymograms. Unlike the acute conditions of brain injuries, 2D zymography did not detect multiple spots with variants of pI values. Neuroinflammatory responses after acute brain injuries are associated with upregulation of proinflammatory cytokines and cellular adhesion molecules, followed by activation of inflammatory cells [55]. These results suggest that proinflammatory response-induced MMP-9 modification isoforms observed in acute brain injuries may not participate in the chronic state associated with diabetes.

We have shown that a 2D zymography approach can improve separation of different isoforms of gelatinases in both *in vitro* and *in vivo* conditions. In summary, our study demonstrated that 2D zymography is an effective method to separate posttranslational modification isoforms of proteases with variant pI values and to identify the modification isoforms of gelatinases that regulate their enzymatic activity in acute brain injuries, but not in chronic metabolic diseases. Such findings provide insight into the role of gelatinase isoforms in the proteolytic processes of enzymes and reveal the proinflammatory response-induced gelatinase characteristics in neurodegenerative diseases.

## Materials and Methods

Animal protocols were approved by the University of Missouri-Columbia Animal Care and Usage Committee. Animals were anesthetized with 2.0% gaseous isoflurane in a nitrogen/oxygen mixture inside a sealed anesthesia chamber. Animals were sacrificed under an overdose of isoflurane.

### Cell Cultures

Human fibrosarcoma HT1080 cell line was from ATCC (Manassas, VA) [13], and the immortalized mouse BV-2 microglial cells were originally from Dr. R. Donato (University of Perugia, Italy) [56]. HT1080 cells (1.0 x 10^5^) were plated into per 35-mm dish containing poly-l-lysine-coated 12-mm glass cover-slips and cultured in DMEM with 10% fetal bovine serum (FBS). BV-2 cells were cultured in DMEM containing 5% heat-inactivated FBS. Both lines were maintained at 37°C in a saturated humidity atmosphere containing 95% air and 5% CO_2_. For *in vivo* activation of microglial cells, BV-2 cells were cultured with 5% FBS. At 70–80% confluence, BV-2 cells were starved with no serum medium for 4 h and treated with 100 ng/ml or 500 ng/ml LPS in the conditioned medium for 16 h.

### Experimental Models of Focal Cerebral Ischemia

Following the protocols approved by the University of Missouri-Columbia Animal Care and Use Committee, C57BL/6J mice weighing 25–30 g were housed in a 12 h light/dark cycle and permitted food and water intake *ad libitum*. Body temperature is maintained at 37°C. The filament-induced transient MCAo was performed as described previously [13,24]. Briefly, a silicon-coated 6–0 monofilament was introduced from external carotid artery into the circle of Willis under isoflurane anesthesia to block the origin of the MCA. After a 90-minute occlusion, the filament was then removed for reperfusion 24 h [13,24,47]. A laser Doppler flowmeter (Moor Laboratory, London, UK) with the probe fixed on the skull surface (3 mm lateral to midline and 2 mm posterior to the Bregma), located at the distal arterial supply of the middle cerebral artery to measure regional cerebral blood flow. The initial reading of regional cerebral blood flow was assigned a value of 100%, and subsequent readings were expressed relative to this value. Mice were sacrificed with an overdose of isoflurane and transcardially perfused with phosphate-buffered saline (PBS) to remove intravascular blood; brains were rapidly removed.

### Experimental Models of Traumatic Brain Injury

The TBI procedure employed an electromagnetic (EM) impactor for CCI and was conducted as previously described [48,57]. Briefly, 8–10 week-old adult male C57BL/6J mice (The Jackson Laboratory, Bar Harbor, ME) weighing 20–25 g were anesthetized with 2.0% gaseous isoflurane in a nitrogen/oxygen mixture inside a sealed anesthesia chamber. Each mouse was then stably placed on a Kopf stereotaxic apparatus (David Kopf Instruments, Tujunga, CA). Body temperature was monitored with a rectal thermistor probe (TH-10Kmp, Cell MicroControls, Norfolk, VA), and maintained at a constant 37°C on a silicon heating pad (HS-3x2.5 Heater, Cell MicroControls, Norfolk, VA). Following a midline skin incision and removal of connective tissue under sterile conditions, a 5.0 mm diameter craniotomy was performed in the left parietotemporal skull using a pedal-operated high-speed micro-drill mounted on the stereotaxic arm. A 5.0-mm diameter bone disc was then removed to expose the left cortex, while keeping the dura mater intact. A MATLAB-controlled EM impactor (Leica Microsystems Impact One, St. Louis, MO) with a 3.0-mm diameter tip was centered at 2.7 mm to the left of the midline suture and 3.0 mm rostral to lambda, at an angle of 15° with the vertical. Once the position was set, the EM impactor delivered a CCI with a velocity of 5.0 m/s and dwell time of 100 ms, at a depth of 2.5 mm. This operation produces a moderately severe contusion in the left parietotemporal cortex and the underlying hippocampus as marked by pronounced behavioral deficits, but virtually no mortality. Following impact, the original skull disc was placed back over the exposed cortex, and the incision was sutured. Each mouse was released from anesthesia and placed in an empty cage over a heating pad for recovery. Mice were sacrificed with an overdose of isoflurane and transcardially perfused with PBS to remove intravascular blood; brains were rapidly removed.

### Experimental Models of Diabetic Obesity

Zucker lean and ZO rats were purchased from Charles River, Inc. (Raleigh, North Carolina) and cared for in accordance with National Institutes of Health guidelines. All procedures were approved in advance by the Institutional Animal Care and Use Committee of the University of Missouri. Four groups of rats were used: Zucker lean rats treated with linagliptin, Zucker lean rats not treated with linagliptin, ZO rats treated with linagliptin and ZO rats not treated with linagliptin. Linagliptin (BI 1356; (R)-8-(3-aminopiperidin-1-yl)-7-but-2-ynyl-3-methyl-1-(4-methyl-quinazolin2-ylmethyl)-3,7-dihydro-purine-2,6-dione) was administered orally by mixing drug with rat chow [58]. The final concentration of linagliptin in chow was 83 mg/kg; this concentration of the drug was chosen to achieve a dose and plasma level of approximately 4 mg/kg/day and 100 nM, respectively.

### 1D and 2D zymography

At 70–80% confluence, conditioned medium of HT1080 cells was collected, washed, and incubated with gelatin-Sepharose 4B (gelatin 4B, GE Healthcare Bio-Sciences, Piscataway, NJ) at 4°C overnight. Different concentrations of APMA were added to the gelatin 4B beads and incubated at room temperature with rotation for 2 or18 h, respectively. Gelatinases in brain homogenates were extracted and detected by 1D zymography as described previously [48]. Densitometry was measured using ImageJ software for the mean intensity of each gelatinolytic band.

In 2D zymography, after incubation with gelatin 4B, gelatinases were released from the beads using rehydration buffer (8 M urea and 4% CHAPS) at room temperature for 40 minutes. Dehydrated immobiline dry strips were swelled with the protein sample for 12 h under 50 V passive rehydration overnight. Proteins were separated by IEF using the following conditions: 250 V for 250 Vh; 500 for 500 Vh,1000 for1000 Vh, gradient to 5000 V for 10000 Vh, and 5000 V for 20000 Vh. Following 1D electrophoresis the strips are equilibrated for 2 x 15 min in re-equilibration buffer, and then placed on gelatin (0.1% gelatin) SDS-PAGE gels (10%), embedded in the agarose (1%) overlay. In order to compare different conditions, after IEF focusing, different strips were cut and applied on the same gelatin SDS-PAGE gel. Purified gelatinases (WBC018, R&D Systems, Inc., Minneapolis, MN and PF038, EMD Millipore, Billerica, MA) or tissue samples were applied on the side of the same gelatin as internal controls. Gelatin zymography electrophoresis was in 120 V for 8 h. After electrophoresis, the gels were washed with 2.5% Triton-X-100 and incubated with developing buffer for 24 h at 37°C and then stained with Coomassie blue.

### Protocol for Dephosphorylation

After treating BV-2 cells with LPS overnight, conditioned medium was incubated with gelatin 4B with or without 10 units of calf intestinal alkaline phosphatase (M182A, Promega, Madison, WI) for 1 h at 37°C in 50 mM Tris-HCl (pH 9.3), 1 mM MgCl_2_, 0.1 mM ZnCl_2_ as previously described [59]. Gelatinases were released from the gelatin 4B beads using rehydration buffer (8 M urea and 4% CHAPS) at room temperature for 40 min. Samples were then analyzed by 2D zymography as described above.

### Immunohistochemistry

Brains were cut in coronal sections for immunohistochemical staining. Briefly, mice were transcardially perfused with 4% paraformaldehyde and brains were dissected and preserved for 24 h in the same buffer. Serial coronal sections (40 μm) were obtained with a vibratome (VT1200S, Leica Microsystems, Inc., Bannockbum, IL). In most instances, a total of 150~160 40-μm tissue sections from each brain were collected into 24-well plates. Immunohistochemistry was carried out on brain sections for microglia with the antibody against ionized calcium-binding adapter molecule 1 (Iba-1), neurons with microtubule-associated protein 2 (MAP-2) as well as NADPH oxidase complex (p47phox). Briefly, fixed coronal sections from the area of interest were washed with phosphate-buffered saline (PBS) and permeabilized with 1% Triton X-100 in PBS for 30 min. Sections were incubated with 5% normal goat serum in 0.05% Triton X-100 in PBS for 60 min, and then overnight with 0.5% normal goat serum in PBS containing the primary antibodies (Iba-1, 1:500, Wako; MAP-2, 1:200, Sigma-Aldrich and p47phox, 1:100, Santa Cruz). The next day, sections were washed and incubated in 0.05% Triton X-100 in PBS containing the appropriate fluorophore-conjugated or biotin-conjugated secondary antibodies (1:300; goat anti-mouse IgG-Alexa594, 1:300 goat anti-rabbit IgG-Alexa488, and 1:500 goat anti-rabbit biotin, Life Technologies/Invitrogen, San Diego, CA) for 2 h, and counterstained in a solution of 1:1000 Hoechst dye 33342 (Life Technologies/Invitrogen, San Diego, CA) or 3,3′-diaminobenzidine (Sigma-Aldrich, St. Louis, MO). Photomicrographs of the areas of interest were captured by a Leica DMI 6000B automated epifluorescence microscope (Leica Microsystems Inc., Buffalo Grove, IL).

### Statistical analysis

Data are expressed as mean values ± SEM and were analyzed by unpaired one-tailed Student's *t*-test. Differences were considered significant at *p*<0.05 for all analyses.

## Supporting Information

S1 Fig Analysis of purified MMP-9 comparing 1D and 2D zymography. Purified MMP-9 was applied for 1D (1 ng) and 2D (2 ng) gelatin zymography. Transparent spots (2D) and bands (1D, left side of the gel) revealed MMP-9 proteolytic activity. Representative 2D zymogram showed a 92-kDa proMMP-9 single spot with pI value between 3 and 4, and a 55-kDa MMP-9 fragment spot with pI value between 4 and 5, corresponding to the respective molecular weights of the bands resolved by 1D zymography on the left of the same gel.
(TIF)

## References

[1] Tang J, Liu J, Zhou C, Alexander JS, Nanda A, Granger DN, et al Mmp-9 deficiency enhances collagenase-induced intracerebral hemorrhage and brain injury in mutant mice. J Cereb Blood Flow Metab. 2004;24(10):1133–45. 15529013 10.1097/01.WCB.0000135593.05952.DE

[2] Rosenberg GA. Matrix metalloproteinases and their multiple roles in neurodegenerative diseases. Lancet Neurol. 2009;8(2):205–16. 10.1016/S1474-4422(09)70016-X 19161911

[3] Yong VW. Metalloproteinases: mediators of pathology and regeneration in the CNS. Nat Rev Neurosci. 2005;6(12):931–44. 16288297 10.1038/nrn1807

[4] Kessenbrock K, Plaks V, Werb Z. Matrix metalloproteinases: regulators of the tumor microenvironment. Cell. 2010;141(1):52–67. 10.1016/j.cell.2010.03.015 20371345 PMC2862057

[5] Asahi M, Wang X, Mori T, Sumii T, Jung JC, Moskowitz MA, et al Effects of matrix metalloproteinase-9 gene knock-out on the proteolysis of blood-brain barrier and white matter components after cerebral ischemia. J Neurosci. 2001;21(19):7724–32. 11567062 10.1523/JNEUROSCI.21-19-07724.2001 PMC6762894

[6] Tsioufis C, Bafakis I, Kasiakogias A, Stefanadis C. The role of matrix metalloproteinases in diabetes mellitus. Curr Top Med Chem. 2012;12(10):1159–65. 22519446 10.2174/1568026611208011159

[7] Yim HE, Ha KS, Bae IS, Yoo KH, Hong YS, Lee JW. Postnatal early overnutrition dysregulates the intrarenal renin-angiotensin system and extracellular matrix-linked molecules in juvenile male rats. J Nutr Biochem. 2012;23(8):937–45. 10.1016/j.jnutbio.2011.04.020 21752621

[8] Opdenakker G, Van den Steen PE, Van Damme J. Gelatinase B: a tuner and amplifier of immune functions. Trends Immunol. 2001;22(10):571–9. 11574282 10.1016/s1471-4906(01)02023-3

[9] Opdenakker G, Nelissen I, Van Damme J. Functional roles and therapeutic targeting of gelatinase B and chemokines in multiple sclerosis. Lancet Neurol. 2003;2(12):747–56. 14636780 10.1016/s1474-4422(03)00587-8

[10] Van den Steen PE, Proost P, Grillet B, Brand DD, Kang AH, Van Damme J, et al Cleavage of denatured natural collagen type II by neutrophil gelatinase B reveals enzyme specificity, post-translational modifications in the substrate, and the formation of remnant epitopes in rheumatoid arthritis. FASEB J. 2002;16(3):379–89. 11874987 10.1096/fj.01-0688com

[11] Labrie M, St-Pierre Y. Epigenetic regulation of mmp-9 gene expression. Cell Mol Life Sci. 2013;70(17):3109–24. 10.1007/s00018-012-1214-z 23184252 PMC11113588

[12] Okamoto T, Akaike T, Sawa T, Miyamoto Y, van der Vliet A, Maeda H. Activation of matrix metalloproteinases by peroxynitrite-induced protein S-glutathiolation via disulfide S-oxide formation. J Biol Chem. 2001;276(31):29596–602. 11395496 10.1074/jbc.M102417200

[13] Gu Z, Kaul M, Yan B, Kridel SJ, Cui J, Strongin A, et al S-nitrosylation of matrix metalloproteinases: signaling pathway to neuronal cell death. Science. 2002;297(5584):1186–90. 12183632 10.1126/science.1073634

[14] Choi SH, Aid S, Kim HW, Jackson SH, Bosetti F. Inhibition of NADPH oxidase promotes alternative and anti-inflammatory microglial activation during neuroinflammation. J Neurochem. 2012;120(2):292–301. 10.1111/j.1471-4159.2011.07572.x 22050439 PMC3386526

[15] Chuang DY, Cui J, Simonyi A, Engel VA, Chen S, Fritsche KL, et al Dietary Sutherlandia and Elderberry Mitigate Cerebral Ischemia-Induced Neuronal Damage and Attenuate p47phox and Phospho-ERK1/2 Expression in Microglial Cells. ASN Neuro. 2014;6(6). 10.1177/1759091414562107 25324465 PMC4271764

[16] Kettenmann H. Neuroscience: the brain's garbage men. Nature. 2007;446(7139):987–9. 17410127 10.1038/nature05713

[17] Chen Z, Shin D, Chen S, Mikhail K, Hadass O, Tomlison BN, et al Histological quantitation of brain injury using whole slide imaging: a pilot validation study in mice. PLoS One. 2014;9(3):e92133 10.1371/journal.pone.0092133 24637518 PMC3956884

[18] Sheng W, Zong Y, Mohammad A, Ajit D, Cui J, Han D, et al Pro-inflammatory cytokines and lipopolysaccharide induce changes in cell morphology, and upregulation of ERK1/2, iNOS and sPLA(2)-IIA expression in astrocytes and microglia. J Neuroinflammation. 2011;8:121 10.1186/1742-2094-8-121 21943492 PMC3206447

[19] Lee EJ, Kim HS. The anti-inflammatory role of tissue inhibitor of metalloproteinase-2 in lipopolysaccharide-stimulated microglia. J Neuroinflammation. 2014;11:116 10.1186/1742-2094-11-116 24970341 PMC4091675

[20] Chen H, Kim GS, Okami N, Narasimhan P, Chan PH. NADPH oxidase is involved in post-ischemic brain inflammation. Neurobiol Dis. 2011;42(3):341–8. 10.1016/j.nbd.2011.01.027 21303700 PMC3079796

[21] Suh SW, Gum ET, Hamby AM, Chan PH, Swanson RA. Hypoglycemic neuronal death is triggered by glucose reperfusion and activation of neuronal NADPH oxidase. J Clin Invest. 2007;117(4):910–8. 17404617 10.1172/JCI30077 PMC1838937

[22] Sun GY, Horrocks LA, Farooqui AA. The roles of NADPH oxidase and phospholipases A2 in oxidative and inflammatory responses in neurodegenerative diseases. J Neurochem. 2007;103(1):1–16. 17561938 10.1111/j.1471-4159.2007.04670.x

[23] Hawkins BT, Davis TP. The blood-brain barrier/neurovascular unit in health and disease. Pharmacol Rev. 2005;57(2):173–85. 15914466 10.1124/pr.57.2.4

[24] Gu Z, Cui J, Brown S, Fridman R, Mobashery S, Strongin AY, et al A highly specific inhibitor of matrix metalloproteinase-9 rescues laminin from proteolysis and neurons from apoptosis in transient focal cerebral ischemia. J Neurosci. 2005;25(27):6401–8. 16000631 10.1523/JNEUROSCI.1563-05.2005 PMC6725288

[25] Jia F, Pan YH, Mao Q, Liang YM, Jiang JY. Matrix metalloproteinase-9 expression and protein levels after fluid percussion injury in rats: the effect of injury severity and brain temperature. J Neurotrauma. 2010;27(6):1059–68. 10.1089/neu.2009.1067 20233042

[26] Launer LJ. Diabetes: vascular or neurodegenerative: an epidemiologic perspective. Stroke. 2009;40(3 Suppl):S53–5. 10.1161/STROKEAHA.108.533075 19064803 PMC7457445

[27] Chen J, Cui X, Zacharek A, Cui Y, Roberts C, Chopp M. White matter damage and the effect of matrix metalloproteinases in type 2 diabetic mice after stroke. Stroke. 2011;42(2):445–52. 10.1161/STROKEAHA.110.596486 21193743 PMC3108495

[28] Kusano K, Miyaura C, Inada M, Tamura T, Ito A, Nagase H, et al Regulation of matrix metalloproteinases (MMP-2, -3, -9, and -13) by interleukin-1 and interleukin-6 in mouse calvaria: association of MMP induction with bone resorption. Endocrinology. 1998;139(3):1338–45. 9492070 10.1210/endo.139.3.5818

[29] Sariahmetoglu M, Crawford BD, Leon H, Sawicka J, Li L, Ballermann BJ, et al Regulation of matrix metalloproteinase-2 (MMP-2) activity by phosphorylation. FASEB J. 2007;21(10):2486–95. 17435175 10.1096/fj.06-7938com

[30] Vandooren J, Geurts N, Martens E, Van den Steen PE, Opdenakker G. Zymography methods for visualizing hydrolytic enzymes. Nat Methods. 2013;10(3):211–20. 10.1038/nmeth.2371 23443633

[31] Visse R, Nagase H. Matrix metalloproteinases and tissue inhibitors of metalloproteinases: structure, function, and biochemistry. Circ Res. 2003;92(8):827–39. 12730128 10.1161/01.RES.0000070112.80711.3D

[32] Vandooren J, Van den Steen PE, Opdenakker G. Biochemistry and molecular biology of gelatinase B or matrix metalloproteinase-9 (MMP-9): the next decade. Crit Rev Biochem Mol Biol. 2013;48(3):222–72. 10.3109/10409238.2013.770819 23547785

[33] Rossano R, Larocca M, Riviello L, Coniglio MG, Vandooren J, Liuzzi GM, et al Heterogeneity of serum gelatinases MMP-2 and MMP-9 isoforms and charge variants. J Cell Mol Med. 2014;18(2):242–52. 24616914 10.1111/jcmm.12181 PMC3930411

[34] Snoek-van Beurden PA, Von den Hoff JW. Zymographic techniques for the analysis of matrix metalloproteinases and their inhibitors. Biotechniques. 2005;38(1):73–83. 15679089 10.2144/05381RV01

[35] Jacob AM, Turck CW. Detection of post-translational modifications by fluorescent staining of two-dimensional gels. Methods Mol Biol. 2008;446:21–32. 10.1007/978-1-60327-084-7_2 18373247

[36] Tyagi SC, Kumar SG, Haas SJ, Reddy HK, Voelker DJ, Hayden MR, et al Post-transcriptional regulation of extracellular matrix metalloproteinase in human heart end-stage failure secondary to ischemic cardiomyopathy. J Mol Cell Cardiol. 1996;28(7):1415–28. 8841929 10.1006/jmcc.1996.0132

[37] Larocca M, Rossano R, Riccio P. Analysis of green kiwi fruit (Actinidia deliciosa cv. Hayward) proteinases by two-dimensional zymography and direct identification of zymographic spots by mass spectrometry. J Sci Food Agric. 2010;90(14):2411–8. 10.1002/jsfa.4100 20672335

[38] Choi NS, Yoo KH, Yoon KS, Maeng PJ, Kim SH. Nano-scale proteomics approach using two-dimensional fibrin zymography combined with fluorescent SYPRO ruby dye. J Biochem Mol Biol. 2004;37(3):298–303. 15469710 10.5483/bmbrep.2004.37.3.298

[39] Serrano SM, Shannon JD, Wang D, Camargo AC, Fox JW. A multifaceted analysis of viperid snake venoms by two-dimensional gel electrophoresis: an approach to understanding venom proteomics. Proteomics. 2005;5(2):501–10. 15627971 10.1002/pmic.200400931

[40] Paes Leme AF, Kitano ES, Furtado MF, Valente RH, Camargo AC, Ho PL, et al Analysis of the subproteomes of proteinases and heparin-binding toxins of eight Bothrops venoms. Proteomics. 2009;9(3):733–45. 10.1002/pmic.200800484 19137556

[41] Wilkesman JG, Schroder HC. Analysis of serine proteases from marine sponges by 2-D zymography. Electrophoresis. 2007;28(3):429–36. 17195259 10.1002/elps.200600332

[42] Lodemel JB, Egge-Jacobsen W, Olsen RL. Detection of TIMP-2-like protein in Atlantic cod (Gadus morhua) muscle using two-dimensional real-time reverse zymography. Comp Biochem Physiol B Biochem Mol Biol. 2004;139(2):253–9. 15465672 10.1016/j.cbpc.2004.08.004

[43] Schulz R. Intracellular targets of matrix metalloproteinase-2 in cardiac disease: rationale and therapeutic approaches. Annu Rev Pharmacol Toxicol. 2007;47:211–42. 17129183 10.1146/annurev.pharmtox.47.120505.105230

[44] Gibbs DF, Shanley TP, Warner RL, Murphy HS, Varani J, Johnson KJ. Role of matrix metalloproteinases in models of macrophage-dependent acute lung injury. Evidence for alveolar macrophage as source of proteinases. Am J Respir Cell Mol Biol. 1999;20(6):1145–54. 10340933 10.1165/ajrcmb.20.6.3482

[45] Warner RL, Bhagavathula N, Nerusu KC, Lateef H, Younkin E, Johnson KJ, et al Matrix metalloproteinases in acute inflammation: induction of MMP-3 and MMP-9 in fibroblasts and epithelial cells following exposure to pro-inflammatory mediators in vitro. Exp Mol Pathol. 2004;76(3):189–95. 15126100 10.1016/j.yexmp.2004.01.003

[46] Candelario-Jalil E, Yang Y, Rosenberg GA. Diverse roles of matrix metalloproteinases and tissue inhibitors of metalloproteinases in neuroinflammation and cerebral ischemia. Neuroscience. 2009;158(3):983–94. 10.1016/j.neuroscience.2008.06.025 18621108 PMC3584171

[47] Cui J, Chen S, Zhang C, Meng F, Wu W, Hu R, et al Inhibition of MMP-9 by a selective gelatinase inhibitor protects neurovasculature from embolic focal cerebral ischemia. Mol Neurodegener. 2012;7:21 10.1186/1750-1326-7-21 22587708 PMC3500265

[48] Hadass O, Tomlinson BN, Gooyit M, Chen S, Purdy JJ, Walker JM, et al Selective inhibition of matrix metalloproteinase-9 attenuates secondary damage resulting from severe traumatic brain injury. PLoS One. 2013;8(10):e76904 10.1371/journal.pone.0076904 24194849 PMC3806745

[49] Hotamisligil GS. Inflammation and metabolic disorders. Nature. 2006;444(7121):860–7. 17167474 10.1038/nature05485

[50] Cai D. Neuroinflammation and neurodegeneration in overnutrition-induced diseases. Trends Endocrinol Metab. 2013;24(1):40–7. 10.1016/j.tem.2012.11.003 23265946 PMC3556486

[51] Hayden MR, Banks WA, Shah GN, Gu Z, Sowers JR. Cardiorenal metabolic syndrome and diabetic cognopathy. Cardiorenal Med. 2013;3(4):265–82. 10.1159/000357113 24474955 PMC3901619

[52] Leonard BL, Watson RN, Loomes KM, Phillips AR, Cooper GJ. Insulin resistance in the Zucker diabetic fatty rat: a metabolic characterisation of obese and lean phenotypes. Acta Diabetol. 2005;42(4):162–70. 16382303 10.1007/s00592-005-0197-8

[53] Brussee V, Guo G, Dong Y, Cheng C, Martinez JA, Smith D, et al Distal degenerative sensory neuropathy in a long-term type 2 diabetes rat model. Diabetes. 2008;57(6):1664–73. 10.2337/db07-1737 18332094

[54] Chander PN, Gealekman O, Brodsky SV, Elitok S, Tojo A, Crabtree M, et al Nephropathy in Zucker diabetic fat rat is associated with oxidative and nitrosative stress: prevention by chronic therapy with a peroxynitrite scavenger ebselen. J Am Soc Nephrol. 2004;15(9):2391–403. 15339988 10.1097/01.ASN.0000135971.88164.2C

[55] Williams AJ, Wei HH, Dave JR, Tortella FC. Acute and delayed neuroinflammatory response following experimental penetrating ballistic brain injury in the rat. J Neuroinflammation. 2007;4:17 17605820 10.1186/1742-2094-4-17 PMC1933533

[56] Shen S, Yu S, Binek J, Chalimoniuk M, Zhang X, Lo SC, et al Distinct signaling pathways for induction of type II NOS by IFNgamma and LPS in BV-2 microglial cells. Neurochem Int. 2005;47(4):298–307. 15955597 10.1016/j.neuint.2005.03.007

[57] Brody DL, Mac Donald C, Kessens CC, Yuede C, Parsadanian M, Spinner M, et al Electromagnetic controlled cortical impact device for precise, graded experimental traumatic brain injury. J Neurotrauma. 2007;24(4):657–73. 17439349 10.1089/neu.2006.0011 PMC2435168

[58] Aroor AR, Sowers JR, Bender SB, Nistala R, Garro M, Mugerfeld I, et al Dipeptidylpeptidase inhibition is associated with improvement in blood pressure and diastolic function in insulin-resistant male Zucker obese rats. Endocrinology. 2013;154(7):2501–13. 10.1210/en.2013-1096 23653460 PMC3689282

[59] Solan JL, Fry MD, TenBroek EM, Lampe PD. Connexin43 phosphorylation at S368 is acute during S and G2/M and in response to protein kinase C activation. J Cell Sci. 2003;116(Pt 11):2203–11. 12697837 10.1242/jcs.00428

