# Supplementary figures and images for: Two-Dimensional Zymography Differentiates Gelatinase Isoforms in Stimulated Microglial Cells and in Brain Tissues of Acute Brain Injuries
Source: PLoS One. 2015 Apr 10;10(4):e0123852. doi: 10.1371/journal.pone.0123852 (PMC4393235; doi:10.1371/journal.pone.0123852)

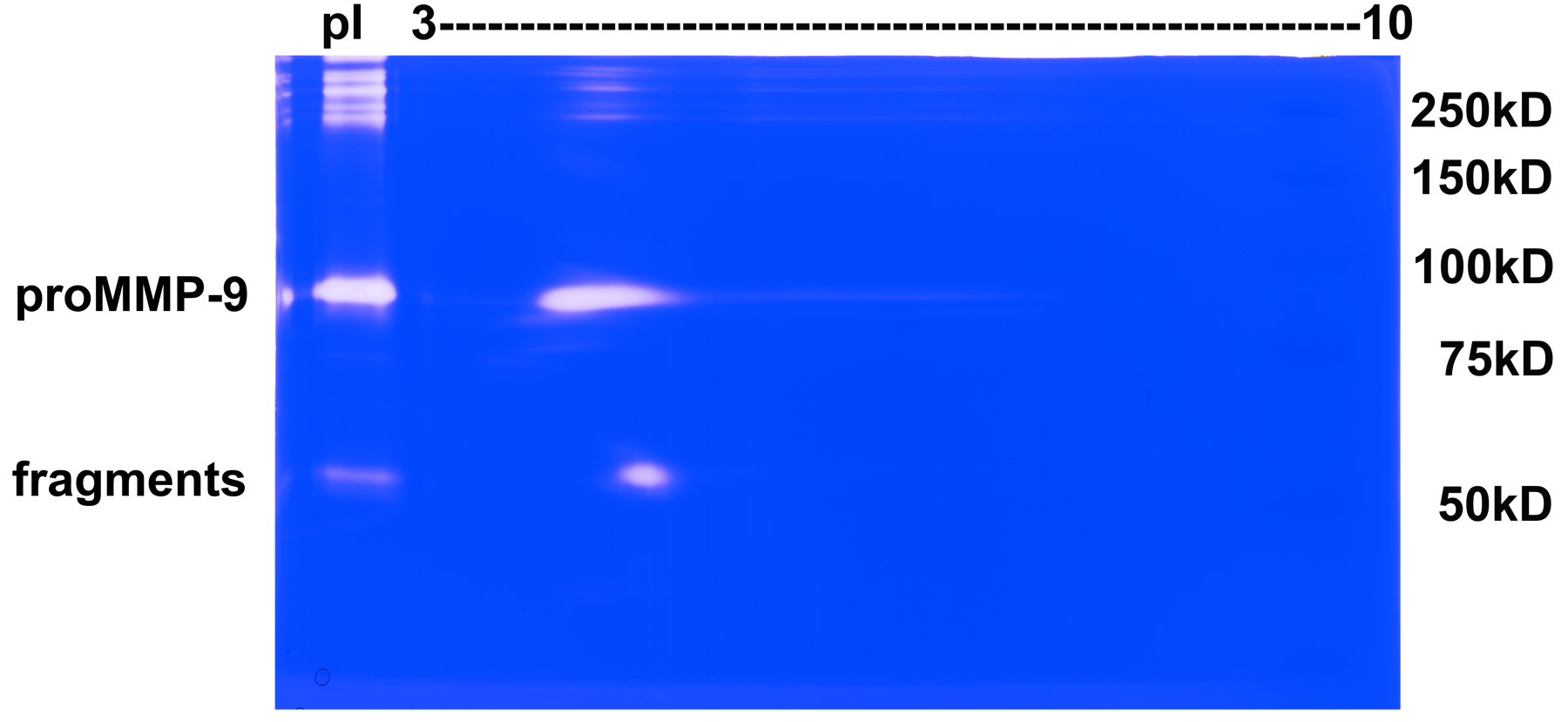

Supplement: S1 Fig — Purified MMP-9 was applied for 1D (1 ng) and 2D (2 ng) gelatin zymography. Transparent spots (2D) and bands (1D, left side of the gel) revealed MMP-9 proteolytic activity. Representative 2D zymogram showed a 92-kDa proMMP-9 single spot with pI value between 3 and 4, and a 55-kDa MMP-9 fragment spot with pI value between 4 and 5, corresponding to the respective molecular weights of the bands resolved by 1D zymography on the left of the same gel. (TIF) [file pone.0123852.s001.tif]
